# Mitigating Water Stress in Plants with Beneficial Bacteria: Effects on Growth and Rhizosphere Bacterial Communities

**DOI:** 10.3390/ijms26041467

**Published:** 2025-02-10

**Authors:** Daniele Nicotra, Alexandros Mosca, Giulio Dimaria, Maria Elena Massimino, Massimiliano Di Stabile, Emanuele La Bella, Farideh Ghadamgahi, Ivana Puglisi, Ramesh Raju Vetukuri, Vittoria Catara

**Affiliations:** 1 Department of Agriculture, Food and Environment, University of Catania, 95123 Catania, Italy; daniele.nicotra@phd.unict.it (D.N.); alexandros.mosca@unict.it (A.M.); giulio.dimaria@unict.it (G.D.); mariaelenamassi@icloud.com (M.E.M.); massimilianodistabile@gmail.com (M.D.S.); emanuele.labella@phd.unict.it (E.L.B.); ipuglisi@unict.it (I.P.); 2 Department of Biomedical and Biotechnological Sciences, University of Catania, 95123 Catania, Italy; 3 Department of Plant Breeding, Swedish University of Agricultural Sciences, 230 53 Alnarp, Sweden; farideh.ghadamgahi@slu.se

**Keywords:** tomato, microbiome, SynCom, bacterial consortia, endophytes, PGPR

## Abstract

Climate change has reshaped global weather patterns and intensified extreme events, with drought and soil salinity negatively impacting the yield and quality of crop production. To mitigate the detrimental effects of drought stress, the introduction of beneficial plant growth-promoting rhizobacteria (PGPR) has proven to be a promising approach. In this study, we evaluated a synthetic microbial community (SynCom) comprising bacterial strains belonging to the species *Bacillus velezensis*, *Pseudomonas simiae*, *P. salmasensis*, *Glutamicibacter halophytocola,* and *Leclercia* sp., which have been demonstrated to promote tomato growth both individually and collectively. The SynCom and most of its individual bacterial strains were shown to mitigate the detrimental effects of polyethylene glycol (PEG)-induced drought stress in vitro in *Arabidopsis thaliana* seedlings, either by reducing alterations in xylem elements or promoting the formation of new xylem strands. In a greenhouse trial, soil drenching with the SynCom and two individual strains, *B. velezensis* PSE31B and *P. salmasensis* POE54, improved the water stress response in soilless-grown tomato plants under a 40% reduced irrigation regime. Additionally, bacterial treatments positively influenced the diversity of rhizosphere bacterial communities, with distinct changes in bacterial composition, which suggest a treatment-specific interplay between the introduced strains and the native microbiome. These findings highlight the potential of microbial consortia and individual PGPR strains as sustainable tools to improve plant resilience to abiotic stresses.

## 1. Introduction

Largely driven by human activities, climate change is increasingly altering global weather patterns, intensifying extreme weather events, and threatening ecosystems, agriculture, and water resources, thus impacting food security [1]. According to recent climate models, non-uniform changes in global hydrological cycles are expected. An increase in annual precipitation is projected for Northern Europe. On the other end, Central Europe and the Mediterranean are likely to experience a significant reduction in the number of rainy days, thus increasing the risk of drought [2]. In these regions, climate change poses a significant threat to water availability, reducing river low flows and annual runoff by 5–70%, which in turn decreases hydropower capacity. In addition, the yields of rainfed crops could decline by up to 64% in certain regions [2].

Drought conditions negatively affect the water potential of crops, compromising their normal physiological and biochemical functions such as photosynthesis, respiration, and nutrient absorption [3,4,5,6,7], also altering root xylem structures [8,9,10,11], thus resulting in a reduction in growth and yield of cultivated plants [12,13].

Plants have evolved a range of mechanisms to cope with water deficits, including hormonal signaling, osmotic adjustment, and stress-responsive gene regulation [14,15,16]. Among these, transcription factors (TFs) play a crucial role in activating drought defense pathways by regulating the expression of stress-related genes [16,17]. Several TF families, including WRKY, CBF (C-repeat binding factor), DREB (dehydration-responsive element binding), MYB, bHLH (basic helix–loop–helix), and ERF (ethylene response factor), have been identified as key players in drought tolerance [17]. These TFs control processes such as stomatal regulation, root architecture modification, antioxidant defense activation, and hormonal balance, all of which are essential for plant survival under drought conditions [16,17]. WRKY transcription factors, for example, are central regulators of plant stress responses. Studies have shown that MbWRKY3, MbWRKY4, and MbWRKY65 from *Malus baccata* enhance drought tolerance by modulating reactive oxygen species (ROS) scavenging and stress-related gene expression [18,19,20]. Similarly, the ethylene response factor MbERF12 has been linked to abscisic acid (ABA)-independent drought tolerance mechanisms [21]. Other key transcription factors, such as MxWRKY53 and MxbHLH18 from *Malus xiaojinensis*, contribute to drought resistance by enhancing root growth and optimizing stomatal responses [22,23].

While these transcription factors are essential for drought adaptation, plants do not operate in isolation. Increasing evidence suggests that their responses can be enhanced by interactions with beneficial microorganisms in the associated microbiome, particularly plant growth-promoting rhizobacteria (PGPR) [24,25]. To mitigate the negative effects of drought, the introduction of beneficial PGPR has emerged as a promising solution [15,26,27]. Also known as rhizobacteria-induced drought endurance and resilience (RIDER), this enhances plant drought tolerance through physiological and biochemical modifications [28]. The underlying physiological mechanisms include the modification of phytohormone (e.g., abscisic acid, salicylic acid, auxin, and gibberellin) levels and antioxidant defense systems. The mechanisms associated with metabolic adjustments, on the other hand, include the accumulation of various organic solutes (such as amino acids, sugars, and polyamines) as well as the production of dehydrins and volatile organic compounds (VOCs) [26,28,29]. In addition, the direct role of PGPR has been attributed to the production of abscisic acid, cytokinin, gibberellic acid, indole-3-acetic acid, trehalose, 1-aminocyclopropane-1-carboxylate (ACC) deaminase, VOCs, amines, and exopolysaccharides [30,31,32,33]. Some PGPR strains have also been shown to activate WRKY, MYB, DREB, and ERF transcription factors, strengthening the plant’s ability to withstand drought [34].

Environmental stressors like drought can alter the plant’s associated microbiome, influencing the plant’s ability to cope with the stress [35]. Water-limiting conditions directly disturb the soil ecosystem by decreasing soil moisture content and inducing changes in the physical and chemical properties of the soil, which eventually affect the soil microbiome [36]. In addition, drought stress indirectly affects plant metabolism and root exudates by altering water absorption by plants, which in turn influences the rhizosphere microbiome [35,36]. Drought can have hereditary effects on soil microbial communities [37,38]. In the last decade, the composition of the root-associated microbiome has been examined under drought stress in many crop species [38,39,40,41].

Tomato (*Solanum lycopersicum* L.) is one of the most important, widespread, and most commonly cultivated horticultural species worldwide and the most cultivated solanaceous crop in the world after potato [42]. Global tomato productivity is threatened by biotic and abiotic stress factors, of which drought and salinity are two of the main environmental stresses that limit the yield and quality of the crop [43,44,45].

On average, the cultivation of tomatoes requires approximately 215 L of water per kilogram [46]. Tomato plants are extremely sensitive to water deficits, which lead to a reduction in seed germination and development, vegetative growth, and reproduction [43,47]. A lack of water has several effects on the biochemical processes in tomato species. It leads to a significant reduction in the relative water content (RWC) and a decrease in photosynthesis and stomatal conductance, with decreased levels of chlorophyll [47]. Water deficits also induce oxidative stress in tomato, with increases in hydrogen peroxide (H_2_O_2_) and superoxide (O_2_^−^) contents as well as malondialdehyde (MDA), which is a biomarker of lipid peroxidation [48]. Tomato plants generate osmolytes such as proline in their cytoplasm, which help to adjust osmotic pressure and reduce the effects of oxidative stress [49]. The sugar content is also involved in the osmoprotectant function under stress conditions, with an increase in the content of soluble sugars, which contributes to the optimal state of the leaf tissue under water stress [48,50].

Some studies have investigated the role of rhizobacteria in tomato protection from drought conditions, and examples of the effective application of individual bacterial strains as well as consortia have been reported [32,51,52,53]. Bacterial inoculants have been shown to mitigate drought stress by altering different physico-biochemical and molecular parameters and improving plant growth attributes. This phenomenon is correlated with the expression of genes involved in pathways related to abscisic acid, osmoprotectant compounds, and heat shock proteins [32,51,53,54].

In our previous research, we selected endophytic bacteria from tomato roots and seeds based on the core microbiome, and identified ten strains with potential as PGPR and biocontrol agents (BCAs) [55]. Additionally, these ten strains, when combined into three distinct synthetic communities (SynComs), demonstrated the ability to promote tomato seedling growth while also influencing the rhizosphere microbiome [56]. Building on these findings, in this study of two different plant models, we evaluated the efficacy of the SynCom that showed the greatest impact on the microbiome, namely ‘MIX2’, in mitigating the water stress response. MIX2 was composed of six strains belonging to *Bacillus velezensis*, *Pseudomonas simiae*, *P. salmasensis*, *Glutamicibacter halophytocola,* and *Leclercia* sp. [56]. Specifically, MIX2 and most of its individual bacteria strains mitigated the detrimental effects of polyethylene glycol (PEG)-induced drought stress in vitro in *Arabidopsis thaliana* seedlings, either by reducing alterations in xylem elements or promoting the formation of new xylem strands.

In a greenhouse trial conducted on soilless-grown tomato plants, soil drenching with MIX2 and two individual strains, *Bacillus velezensis* PSE31B and *Pseudomonas salmasensis* POE54, improved the water stress response induced by a 40% reduction in the irrigation regime. Additionally, bacterial treatments increased the diversity of the rhizosphere bacterial community, with distinct changes in bacterial composition depending on the specific treatment applied.

## 2. Results

### 2.1. Bacterial Treatments Affect Root Xylem Structure in Arabidopsis thaliana Under Drought Stress

**Figure 1:**
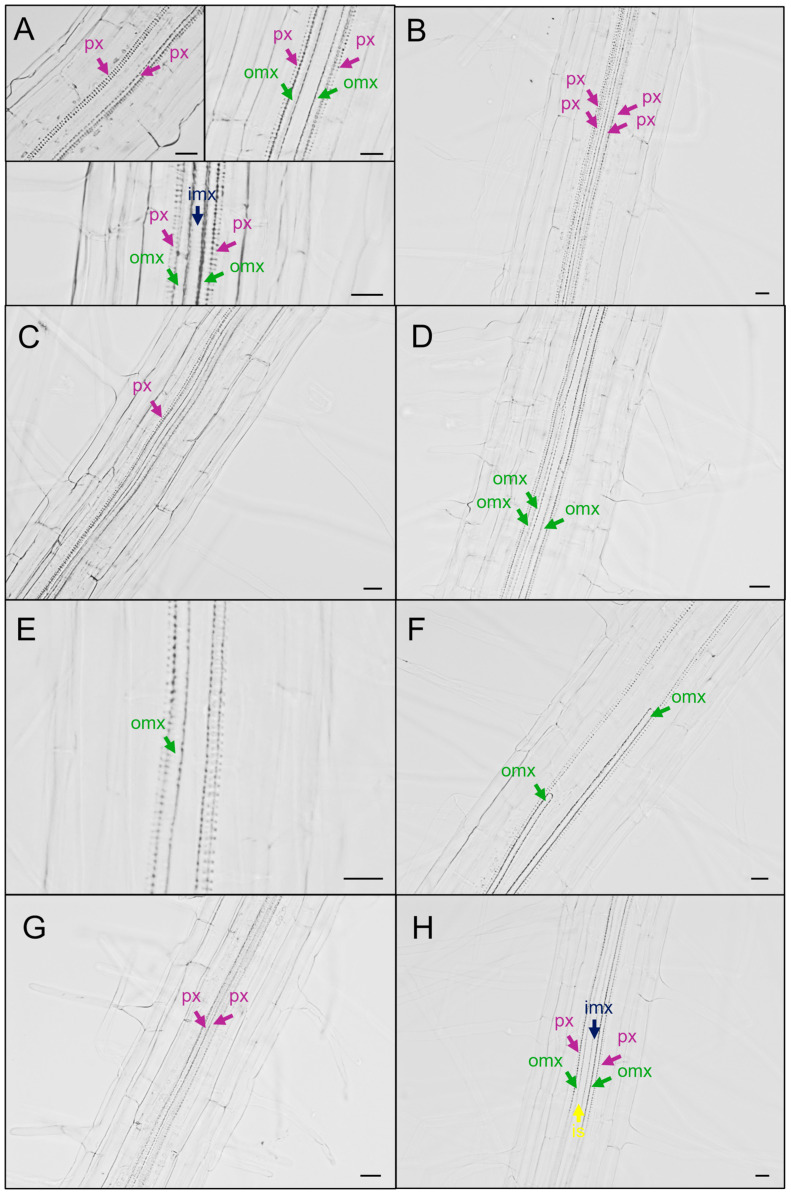
Root xylem phenotypes assigned to eight classes: class 1 (**A**), typical root xylem morphology, with two protoxylem strands close to the root tip (**upper left**); two outer metaxylem strands adjacent to the protoxylem (**upper right**) at the center of the root; the inner metaxylem strand replacing the inner space close to the collar (**bottom**); class 2, additional protoxylem strands (**B**); class 3, fewer protoxylem strands (**C**); class 4, additional metaxylem strands (**D**); class 5, fewer metaxylem strands (**E**); class 6, discontinuous xylem strands (**F**); class 7, absence of the inner space close to the root tip (**G**); class 8, inner space close to the root collar (**H**). Px, protoxylem; Omx, outer metaxylem; Imx, inner metaxylem; Is, inner space. Scale bars = 10 µm.

The efficacy of seed treatment with SynCom MIX2 and its individual strains (*B. velezensis* PSE31B, *B. velezensis* PFE42, *P. salmasensis* POE54, *P. simiae* POE78A, *Glutamicibacter halophytocola,* and *Leclercia* sp. S52) was evaluated through microscopy observations of the root xylem in seven-day-old *A. thaliana* seedlings exposed to drought stress simulated with PEG-infused agar plates. Based on microscope observations, eight classes of root xylem phenotypes were identified based on Ramachandran et al. (2020) [57], with some modifications (Figure 1). The different phenotypes observed are shown in Supplementary Figure S1.

The root xylem of *Arabidopsis* seedlings not exposed to PEG showed a typical morphology [58]: close to the root tip, two protoxylem strands separated by the inner space; the central part of the root, two outer metaxylem strands between the protoxylem vessels; close to the collar, an inner metaxylem vessel replacing the inner space (Figure 1A). Protoxylem vessels, which have annular and spiral thickening, are formed first during the early stages of growth, while metaxylem vessels, which usually have reticulated and pitted thickening, are formed later during development [58].

**Figure 2:**
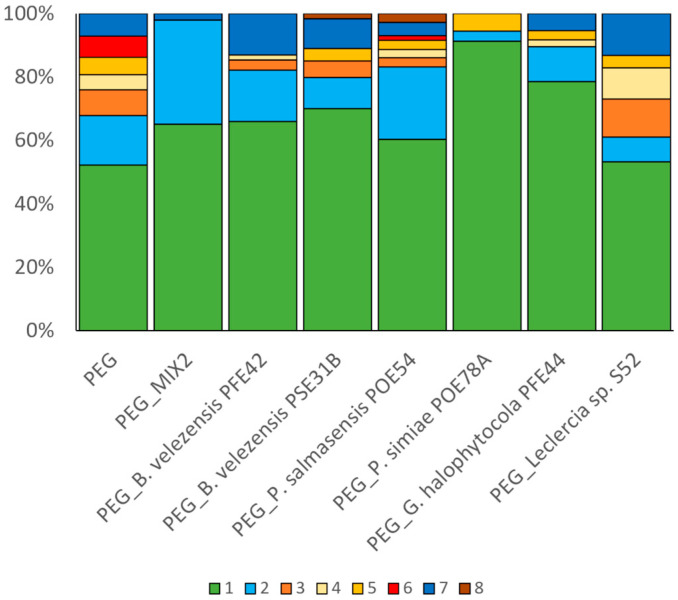
Frequency of xylem phenotypes observed in the roots of *A. thaliana* seedlings exposed to drought stress using PEG-infused plates. Approximately 10 observations were conducted for each seedling to cover the entire length of the primary root. Three plates with eight seedlings each were used per treatment.

Phenotypes were categorized, and the number of observations showing a certain phenotype were used to calculate their frequency. Only 52% of the microscope observations of PEG-exposed *Arabidopsis* seedlings showed the typical xylem morphology (class 1), and the changes observed were assigned to seven classes (Figure 1 and Figure 2). The most common class was the presence of extra protoxylem strands (class 2, 15.6%), followed by a reduction in protoxylem strands (class 3, 8.1%), the absence of the inner space close to the root tip (class 7, 7.0%), discontinuous xylem strands (class 6, 6.7%), and the presence of extra or fewer metaxylem strands (class 4, 4.8% and 5, 5%, respectively) (Figure 1 and Figure 2).

In addition, tyloses (Supplementary Figure S2A) and circular bodies (Supplementary Figure S2B) next to the protoxylem vessels were also observed.

The predominance of a typical root morphology was recorded for PEG-exposed seedlings obtained from seeds treated with MIX2 or the individual strains, ranging from 53% (*Leclercia* sp. S52) to 91% (*P. simiae* POE78A) of the observations (Figure 2). The production of extra protoxylem strands, which is linked to an increased tolerance to drought stress [57], was most observed in the roots of the MIX2 treatments (33% of the observations) and POE54 (23% of the observations) seedlings. In the roots of seedlings from POE54-treated seeds, all the classes of phenotypes described were observed, and together they accounted for 17% of the observations (Figure 2).

### 2.2. Bacterial Treatments Mitigated the Impact of the Reduction in Water Supply in Soilless-Grown Tomatoes

#### 2.2.1. Growth and Yield Attributes

The SynCom MIX2 and two of its strains that appeared to promote growth in tomato [55], namely *Bacillus velezensis* PSE31B and *Pseudomonas salmasensis* POE54, were tested in a greenhouse trial (Supplementary Figure S3). Tomato plants were grown soilless in coconut fiber bags, and bacteria were applied twice, before transplanting by submerging the roots in the suspension and by soil drenching two weeks later. The plants were subjected to a 40% reduction in the irrigation regime 24 h after the second bacterial treatment. Water stress (WS) affected the growth of tomato plants in the greenhouse, significantly decreasing plant height (*p* < 0.01) and stem diameter (*p* < 0.001) compared to control plants, as well as the dry matter, although not significantly (*p* = 0.219) (Table 1). The growth rate between the time-points T0-T1, i.e., before and after the stress application, and T8, i.e., the end of the trial, was lower in WS than in control plants (*p* < 0.05) (Table 1). Belowground plant growth could not be assessed because the roots were deeply intertwined with the coconut fiber. However, notable differences were observed between the control and the WS plants, with many rootlets exhibiting necrosis. In contrast, the roots of the bacterial-treated plants were more similar to those of the control (Supplementary Figure S4).

The percentage of flowering plants in the different trusses was also affected by the water deficit, with lower values in stressed plants compared to the non-stressed controls (Table 2). Although the data were not significant, the difference increased progressively with the number of trusses over time. Compared with that of the control plants, the number of fruits in the first truss of the WS plants was greater, whereas the number of fruits in the second and third trusses was lower (Table 2).

The plant height and growth rate of plants inoculated with MIX2 (WS_MIX2), as well as the individual strains *Bacillus velezensis* PSE31B (WS_PSE31B) and *Pseudomonas salmasensis* POE54 (WS_POE54) exposed to the stress, differed significantly (*p* < 0.01 and *p* < 0.05, respectively) from those of the WS plants (Table 2). Conversely, the stem diameter was similar in bacteria-treated and untreated plants exposed to water stress (Table 2). The bacterial treatments increased the dry matter of the stressed plants, which also presented higher values than the control plants in some cases; however, these differences were not significant (*p* = 0.219) (Table 2). Although the yield values were not significant, a positive trend of the bacterial treatments was found in ensuring higher percentages of flowering plants. The fruit yield was overall lower in bacteria-treated plants compared to non-stressed controls (Table 2). Compared with those in WS, the number of marketable fruits in the first truss and the total number of fruits in the second truss slightly increased in WS_PSE31B plants (Table 2). The fruit weight of the PSE31B-treated plants was greater than that of the WS-treated plants in all trusses (Table 2).

#### 2.2.2. Quality Parameters of Tomato Fruits

The analysis of the fruit quality parameters highlighted significant differences between fruits from the control plants and tomato plants exposed to a reduced volume of irrigation water (Table 3). Regardless of the bacterial treatments, the stressed plants showed a tendency to increase the fruit firmness compared to the controls, and significant values were recorded in WS_POE54 and WS_PSE31B (*p* < 0.0001). A significant increase in juice titratable acidity (*p* = 0.020) and a consequent decrease in the sugar–acid ratio (*p* = 0.029) in fruits from WS_PSE31B and WS_MIX2 were observed (Table 3). The total soluble solid (TSS) content of the juice was not significantly affected.

### 2.3. Effects of Bacterial Treatments on the Resident Rhizosphere Microbiome of Water-Stressed Tomato Plants

#### 2.3.1. Richness and Diversity of Bacterial Communities

**Figure 3:**
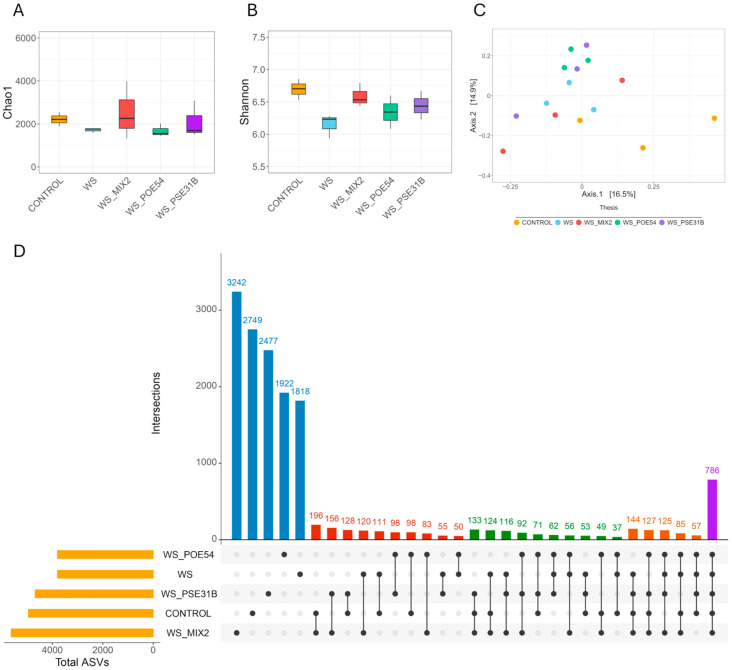
α diversity of the bacterial communities using Chao1 richness (**A**) and Shannon diversity (**B**) indices, respectively. (**C**) PCoA plots depicting the β diversity of the bacterial communities based on the Bray–Curtis dissimilarity matrix. Each point on the graph corresponds to a single sample (biological replication). (**D**) UpSet plot showing the number of detected ASVs in each treatment (horizontal bars), and unique or shared ASVs (individual or connected points, respectively) in and among theses.

The bacterial communities of the rhizosphere of tomato plants grown soilless in coconut fiber bags in the greenhouse were analyzed at the end of the trial. Three replicates for each treatment were analyzed. Illumina sequencing of bacterial 16S rRNA yielded 15,844,266 reads. After quality filtering (length trimming, denoising, exclusion of chimeric sequences, and non-microbial reads), a total of 1,727,426 reads were recovered and assigned to 15,061 Amplicon Sequence Variants (ASVs) (Supplementary Table S1). The bacterial α diversity was estimated by Chao1 richness and Shannon diversity indices. Based on the Chao1 index, higher richness values were observed in the rhizospheres of the control and WS_MIX2 plants compared to the WS and WS_PSE31B and WS_POE54 plants (Figure 3A). Bacteria-treated plants showed higher Shannon diversity values compared to WS plants, with WS_MIX2 showing the highest value (Figure 3B).

In addition, the rhizosphere bacterial communities of the WS_MIX2 and control plants exhibited the highest number of total (5634 and 4948, respectively) and unique (3242 and 2749, respectively) ASVs. The lowest values were found in the rhizosphere of WS plants (3799 and 1818 of total and unique ASVs, respectively) (Figure 3D). A total of 786 ASVs were shared among all the treatments. WS_MIX2 and control plants showed the highest number of common ASVs (196) (Figure 3D). The PCoA for the β diversity based on the Bray–Curtis dissimilarity highlighted that the rhizosphere bacterial communities of the control plants differed from those of the plant rhizospheres exposed to the water stress, either treated or not treated with the bacterial inoculants (PERMANOVA, R^2^ = 0.36, *p*-value = 0.003) (Figure 3C). Among the bacterial treatments, the tomato rhizosphere bacterial communities of WS_POE54 plants clustered separately from the communities of the rhizosphere of the WS plants (Figure 3C).

#### 2.3.2. Relative Abundance of Bacterial Communities

**Figure 4:**
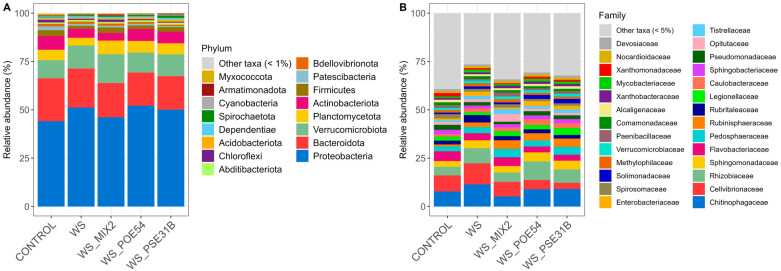
Relative abundances of the bacterial communities at the phylum (**A**) and family (**B**) taxonomic levels in the rhizosphere of control and bacteria-treated and untreated water-stressed plants. Taxa less than 1% and 5% in abundance are reported as “other taxa” for the phylum and family level, respectively.

The dominant bacterial taxa at the phylum level across all treatments were Proteobacteria, Bacteroidota, and Verrucomicrobiota, showing relative abundances > 10% in each sample and accounting for more than 75% of the overall bacterial communities (Figure 4A, Supplementary Table S2). Compared to the control, WS plants exhibited an increased abundance of Proteobacteria (51.2% vs. 44.1%) and Verrucomicrobiota (11.9% vs. 9.4%), and a reduction in Bacteroidota (20.2% vs. 22.2%), Planctomycetota (4.0% vs. 5.4%), Actinobacteriota (4.7% vs. 7.1%), and Firmicutes (1.5% vs. 3.0%), as well as low abundant phyla (<1%) (Figure 4A).

The bacterial communities of the rhizosphere of the plants subjected to water stress but treated with the bacterial inoculants showed a reduction in Bacteroidota (WS_MIX2, 17.6%; WS_PSE31B, 17.3%; WS_POE54, 17.1%) and an increase in Planctomycetota (WS_MIX2, 7.0%; WS_PSE31B, 5.7%; WS_POE54, 6.1%) and Firmicutes (WS_MIX2 2.9%; WS_PSE31B, 2.3%; WS_POE54, 1.9%) compared to WS plants (20.2%, 4.0%, and 1.5%, respectively) (Figure 4A). In addition, WS_MIX2 plants showed a decreased abundance of Proteobacteria compared to WS (46.2% vs. 51.2%), with more similar values to the bacterial communities of the control plants (44.1%), and an increase in Verrucomicrobiota (15.0% vs. 11.9%) (Figure 4A). WS_PSE31B and WS_POE54 showed an increase in Actinobacteriota compared to WS plants (6.1% vs. 4.7%) (Figure 4A).

At the family level, the most abundant bacterial taxa in the rhizosphere across all treatments were Chitinophagaceae (Bacteroidota), Cellvibrionaceae, and Rhizobiaceae (Proteobacteria) (Figure 4B, Supplementary Table S3). WS plants showed higher abundances of these three families (11.4%, 11.0%, and 7.8%, respectively) than control plants (7.8%, 8.2%, and 4.5%, respectively) as well as of Sphingomonadaceae (Proteobacteria) (4.1% vs. 2.9%), Rubinisphaeraceae (Planctomycetota) (2.4% vs. 1.1%), Rubritaleaceae (Verucomicrobiota) (3.8% vs. 2.0%), and Enterobacteriaceae (Proteobacteria) (2.2% vs. 0.9%) (Figure 4B). In turn, a decreased abundance of Legionellaceae (Proteobacteria) (1.4% vs. 2.1%), Sphingobacteriaceae (Bacteroidota) (1.5% vs. 2.3%), Pseudomonadaceae (Proteobacteria) (1.7% vs. 2.5%), and Paenibacillaceae (Firmicutes) (0.8% vs. 1.4%) was observed, as well as low-abundant taxa (<5%) (Figure 4B). The bacterial communities of the rhizosphere of plants treated with the bacterial inoculants exhibited an increase in Rubinisphaeraceae (WS_MIX2, 4.5%; WS_PSE31B, 4.3%; WS_POE54, 3.7%), Legionellaceae (WS_MIX2, 2.4%; WS_SE31B, 3.6%; WS_POE54, 2.2%) and low-abundant taxa (<5%) (WS_MIX2, 34.2%; WS_PSE31B, 32.2%; WS_POE54, 30.7%) compared to WS plants (2.4%, 1.4%, and 26.5%, respectively). A decrease in Chitinophagaceae (WS_MIX2, 5.3%; WS_PSE31B, 9.0%; WS_POE54, 8.9%) and Cellvibrionaceae (WS_MIX2, 7.5%; WS_PSE31B, 3.2%; WS_POE54, 4.8%) was observed in comparison to WS (11.4% and 10.9%, respectively) (Figure 4B). The rhizosphere bacterial communities of WS_MIX2 plants also showed an increase in Flavobacteriaceae (Bacteroidota) (4.5% vs. 3.6%), Pedosphaeraceae (Verrucomicrobiota) (4.3% vs. 3.1%), Opitutaceae (Verrucomicrobiota) (3.9% vs. 1.5%), and Paenibacillaceae (2.0% vs. 0.8%) and a decrease in Rhizobiaceae (4.8% vs. 7.8%), Sphingomonadaceae (3.4% vs. 4.1%), and Enterobacteriaceae (0.8% vs. 2.2%), overall showing a more similar family representativeness to that of the control plants (Figure 4B). WS_PSE31B and WS_POE54 showed a higher relative abundance of Sphingobacteriaceae (Bacteroidota) (2.1% and 1.9% vs. 1.5%) and Pseudomonadaceae (1.9% and 2.2% vs. 1.6%) compared to WS plants. Interestingly, WS_POE54 also showed a higher abundance of Rhizobiaceae (9.6% vs. 7.8%) and WS_PSE31B of Pedosphaeraceae (4.1% vs. 4.3%) (Figure 4B).

#### 2.3.3. Enriched and Depleted ASVs Across the Bacteria-Treated Plants Compared to the Water-Stressed Plants

**Figure 5:**
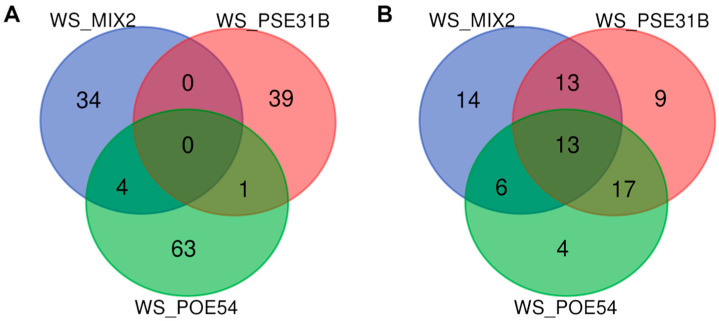
The differential abundance of unique and shared ASVs, highlighting (**A**) enriched and (**B**) depleted groups, in bacteria-treated samples compared to untreated samples exposed to water stress.

The differential abundance analysis was performed at the ASV level, comparing bacteria-treated plants subjected to water stress with WS plants. Venn analysis revealed a marked statistically significant (*p* value < 0.05, False Discovery Rate, FDR) difference in the number of enriched and depleted ASVs across the samples in response to the bacterial inoculation, despite approximately eight weeks having passed since the last bacterial treatment (Figure 5). Specifically, a higher number of unique enriched ASVs in each treatment was observed, while the highest number of depleted ASVs were shared among the bacteria-treated plants.

Regarding the 141 enriched ASVs, 34, 39, and 63 ASVs were unique to WS_MIX2, WS_PSE31B, and WS_POE54, respectively (Figure 5A). In WS_MIX2, the ten most representative ASVs belonged to the phylum Verrucomicrobiota, mostly represented by the genus *Neochlamydia* (Supplementary Table S4), followed by eight ASVs belonging to Proteobacteria and seven ASVs to Bacteroidota. In addition, among the four enriched ASVs of Firmicutes, three belonged to *Paenibacillus* (Supplementary Table S4). Regarding the 39 ASVs enriched in WS_PSE31B, 20 ASVs belonged to the phylum Proteobacteria, and 7 ASVs belonged to Bacteroidota, mostly represented by the *Flavobacterium* genus. The 63 ASVs in WS_POE54 were mostly represented by Proteobacteria, Bacteroidota (14 ASVs in both phyla), and Planctomycetota (13 ASVs).

Overall, 76 ASVs were depleted across all the bacteria-treated plants. Thirteen ASVs were shared among all three treatments (Figure 5B, Supplementary Table S5). Seventeen ASVs were shared between WS_PSE31B and WS_POE54, whereas thirteen and six ASVs were shared by WS_MIX2 and WS_PSE31B and WS_POE54, respectively. As observed in the enriched ASVs, all these ASVs were mostly represented by Proteobacteria, Bacteroidota, and Planctomycetota phyla (Supplementary Table S5).

## 3. Discussion

This study illustrates the potential of SynCom MIX2 and its individual bacterial strains to mitigate water stress in two distinct plant models, *Arabidopsis thaliana* and tomato. MIX2, which was composed of six bacterial strains from the genera *Bacillus*, *Pseudomonas*, *Glutamicibacter*, and *Leclercia*, was selected based on its significant plant growth promotion activity and impact on the plant microbiome [56]. In vitro assay with *A. thaliana* revealed that MIX2 and most of its individual strains alleviated polyethylene glycol (PEG)-induced drought stress by reducing alterations in xylem morphology and, in some cases, strongly promoting the formation of new xylem strands. These structural changes are critical for maintaining hydraulic conductivity under stress conditions and underscore the potential of these strains to improve drought tolerance [57]. In the greenhouse trial, with soilless-grown tomato plants, soil drenched with MIX2 or the individual strains *Bacillus velezensis* PSE31B and *Pseudomonas salmasensis* POE54, which have shown a good capacity to promote plant growth [55], significantly mitigated the detrimental effects of a 40% reduction in irrigation. These treatments not only improved plant performance under drought stress but also positively influenced the diversity of the rhizosphere bacterial communities. Notably, each bacterial treatment induced distinct changes in community composition, thus suggesting that the interplay between the introduced strains and the native microbiome was highly treatment specific.

Drought has become one of the most devastating abiotic stresses affecting cultivated crops [2]. It adversely impacts the water potential of crops, disrupting essential physiological and biochemical processes, including photosynthesis, respiration, and nutrient uptake [3,4,5,6,7], ultimately leading to reduced growth and yield in cultivated plants [12,13]. In our study, we used the model plant *A. thaliana* to evaluate the protective effects of seed treatments with SynCom MIX2 and individual bacterial strains on the tracheary elements of the root xylem of seedlings exposed to PEG-induced drought stress. Water-limiting conditions have been shown to alter xylem structures in different plant species [8,9,10,11]. Several studies have focused on *A. thaliana*, and analyzed how changes in xylem morphology can influence water transport and stress resilience [57,58,59]. Our results suggest that bacterial treatments, either as SynCom or individual strains, can induce modifications in root xylem morphology, which may improve water transport capacity under stress conditions. After 24 h of PEG-induced drought stress, *A. thaliana* seedlings exhibited altered xylem patterns, with MIX2 treatment strongly promoting additional protoxylem strands, while *P. simiae* POE78A preserved normal morphology. In addition, plants treated with PGPR did not show tyloses and circular bodies, which in other studies have been associated with vessel cavitation phenomena and embolism [60,61,62]. These changes align with drought-induced adaptations, such as increased protoxylem, to improve embolism resistance [59,63]. The role of ABA and JA in xylem development suggests that PGPR treatments likely modulate hormonal pathways, thus aiding stress resilience [26,28,29,59,63].

PGPR treatments appeared to be a reliable solution to help crops withstand environmental stressors through multiple mechanisms [15,26,27,29] (Supplementary Figure S5). Drought and salinity are two of the most important abiotic factors that limit tomato production in the Mediterranean basin [29,30]. Tomato is highly sensitive to water deficit conditions, especially in specific developmental stages, which negatively affect both the yield and the quality of the production [43,45].

In the trial conducted under commercial conditions in the greenhouse, a reduction in the irrigation regime of 40% led to a significant decrease in plant height and stem diameter in soilless-grown tomato plants compared to non-stressed control plants. The dry matter showed the same trend, although the decrease was not significant. The reduction in such growth parameters has been commonly observed in tomato plants exposed to drought conditions [47]. In particular, plant height, fresh and dry weights, [48,52,64,65] and stem diameter [52,66] are the growth characteristics most affected by water stress.

Treatments with either the bacterial SynCom or the two individual strains belonging to the genera *Bacillus* and *Pseudomonas* alleviated the effects of the water stress on tomato plant growth, with similar plant height values to those of non-stressed plants. The stem diameter values were lower than those of the control plants but were overall higher than those of water-stressed plants, although the differences were not significant. Under suboptimal irrigation, an increase in dry matter was observed in treated compared to the untreated plants with higher values than those of the control plants for treatments with *B. velezensis* PSE31B and MIX2. Other studies have shown the ability of PGPR to improve plant growth under drought stress. An *Azotobacter–Azosprillium*-based biostimulant mitigated the effects of drought stress, increasing shoot and root fresh and dry weight, plant height, and stem diameter [52]. The seed application of *B. subtilis* RHizo SF 48 significantly enhanced plant growth in tomato plants exposed to different levels of drought stress compared to untreated plants [32]. This ability was attributed to the production of ACC deaminase and to the protection against oxidative damage [32]. Two PGPR belonging to *Pseudochrobactrum* and *Leucobacter* genera applied alone or in consortia (with or without a *Sphingobacterium* strain), increased the height, stem diameter, and the number of leaves of tomato plants under drought stress conditions [53]. Krishna et al. (2022) applied a hexa-PGPM consortium composed of *Bacillus*, *Paenibacillus*, *Pseudomonas,* and *Trichoderma* strains in tomato plants under half irrigation conditions in greenhouse. The consortium ensured lower cellular damage and better plant growth and yield than non-inoculated plants, increasing the plant height and fresh and dry weight, the root length, as well as the number and weight of fruits [51]. A significant increase in shoot fresh weight was also reported by Cirillo et al. (2023) in plants treated with a consortium of *Azotobacter chroococcum* and *Trichoderma afroharzianum* compared to untreated plants exposed to water stress [54].

Although drought stress usually induces early flowering in tomato plants [12,67], we observed a progressive delay in flowering. The opposite trend was instead observed in the tomato plants treated with the bacteria. The fruit weight was lower in plants subjected to a water deficit than non-stressed plants, in line with Rady et al. (2020), Chakma et al. (2021), and Sivakumar and Srividhya (2016) [12,48,65]. Treatment with *B. velezensis* PSE31B resulted in a higher number of marketable fruits and fruit weight compared to stressed plants. An increased number and weight of fruits in plants treated with PGPR under drought stress conditions has been documented [48,51]. However, bacterial treatments were also observed to not affect the yield and number of fruits in tomato plants subjected to a suboptimal irrigation regime [54].

Fruit quality parameters are very important in the tomato industry. Fruit taste is of great importance, with a strong positive correlation observed between increased total sugar and acid levels and flavor acceptability [68].

In our study, the water deficit slightly reduced the total soluble solid content in fruit compared to fruit from plants not exposed to the stress, conversely to findings by Chakma et al. (2021) and Rady et al. (2020) [48,65]. In contrast, fruits from plants subjected to bacterial treatments and exposed to the stress slightly increased TSS compared to the controls. Fruit from plants exposed to water stress showed enhanced fruit firmness compared to non-stressed plants, and fruits from *P. salmasensis* POE54-treated plants showed the highest value. The titratable acidity was slightly lower in fruit from plants exposed to drought conditions than fruit from control plants. On the other hand, the higher values of titratable acidity were measured in fruits from plants treated with *B. velezensis* PSE31B and MIX2, which consequently showed a lower sugar–acid ratio. These effects on acidity level and fruit firmness suggest a positive impact of the bacterial treatments in terms of consumer flavor acceptability and postharvest storage, with low acidity and firmness values being associated with quality loss [68,69].

The application of rhizobacteria not only directly benefits the plants by enhancing stress tolerance but could also positively reshape the plant microbiome, fostering a more resilient and supportive microbial environment [70].

The analysis of bacterial communities showed that the dominant bacterial phyla in the rhizosphere of soilless-grown tomato plants were Proteobacteria, Bacteroidota, and Verrucomicrobiota. This is in line with a study in which the most abundant phyla in the coconut fiber substrate were Proteobacteria, Actinobacteriota, and Firmicutes, while two months after transplanting tomato, the rhizosphere was enriched in Bacteroidota and Verrucomicrobiota [71]. In addition, the study highlighted that the rhizosphere microbiome was mainly shaped by the substrate or soil in which the plants were grown [71].

Drought disturbs the soil ecosystem directly by decreasing the soil moisture content and inducing changes in the physical and chemical properties of the soil, which eventually affect the soil microbiome [36].

The rhizosphere of water-stressed plants showed a reduced α diversity compared to non-stressed plants, also confirmed by the lowest number of total and unique ASVs found in the rhizosphere bacterial communities of plants exposed to water stress. In line with our findings, it has been previously observed that low soil moisture content leads to a reduction in both the abundance and diversity of bacterial communities in some crops [36,72,73] as well as in tomato [74]. In contrast, Gaete et al. (2021) reported an increase in the α diversity of bacterial communities in response to a water deficit in the rhizosphere of a susceptible cultivar but not in a tolerant cultivar [75]. We observed an increased abundance of Proteobacteria and Verrucomicrobiota in water-stressed plants compared to the controls, and conversely a reduced abundance of Bacteroidota, Planctomycetota, Actinobacteriota, and Firmicutes. Among these taxa, Actinobacteriota usually increases under drought conditions, and strains from this phylum have shown an ability to improve drought tolerance [76,77], as found by Gaete et al. (2021) in tomato [75] and also observed in many other plant systems (reviewed by Ye et al., 2022 [36]). Interestingly, in our trial, bacterial treatments partially re-established the composition of bacterial community altered by drought stress, increasing the abundance of Planctomycetota, Firmicutes, and Acidobacteriota compared to water-stressed plants. Compared with untreated water-stressed plants, plants treated with *B. velezensis* PSE31B or *P. salmasensis* WS_POE54 presented an increase in Actinobacteriota. Overall, the α-diversity values of the plants in the MIX2 treatment were similar to those of the non-stressed plants, and the highest values of total and unique ASVs were detected in the MIX2 treatment compared with those in any other treatment.

Differential abundance analysis at the ASV level showed a significant differentiation of enriched and depleted bacterial ASVs in the rhizosphere of bacteria-treated plants compared to water-stressed plants. Each bacterial inoculant specifically recruited unique ASVs, while the majority of depleted ASVs were common between treatments.

The ability of PGPR to restore the diversity of bacterial communities altered by water deficit conditions was also observed by Cirillo et al. (2023) [54], who reported a considerable increase in the abundance and diversity of microbial populations in the rhizosphere of stressed plants treated with a consortium of *Azotobacter chroococcum* and *Trichoderma afroharzianum* compared to untreated plants, with the Shannon diversity index exhibiting higher values, especially within bacterial populations [54].

In our study, a positive effect of the bacterial treatments was observed. For most of the phenotypic traits analyzed, treated plants tended to counteract the negative effects induced by water stress, although not all results were statistically significant. Similar trends have been observed in other greenhouse studies [54]. The growth conditions in commercial environments involve longer timeframes and expose plants to varying environmental factors such as humidity, temperature, and biotic stressors, thus potentially increasing variability among plants in randomized replicates.

Using microbial consortia in agriculture tends to have greater benefits than applying single microbial inoculants. In fact, microbial consortia form a complex network of interactions within the resident microflora and can offer a wider range of beneficial functions, coupling different mechanisms of action [78,79]. The SynCom MIX2 showed no important differences with the bacterial strains tested individually. However, some differences in phenotypic traits were observed in both the model plants which could be attributed to the traits putatively predicted in their genomes [55]. Bacterial treatments, especially MIX2, increased the diversity but also changed the composition of the bacterial communities. The specific enrichment of bacterial ASVs for each of the bacterial treatments was observed, which merits further investigation.

## 4. Materials and Methods

### 4.1. Bacterial Strains Used in This Study

Six bacterial strains were used in this study: *Bacillus velezensis* strains PSE31B and PFE42, *Pseudomonas salmasensis* POE54, *P. simiae* POE78A, *Glutamicibacter halophytocola* PFE44, and *Leclercia* sp. S52 [55]. A SynCom composed of all six strains, namely MIX2, was also tested [56].

### 4.2. Bacterial Inoculum Preparation

Bacterial strains were routinely maintained on Nutrient Agar (NA, Oxoid, Milan, Italy) amended with 1% dextrose (NDA) at 27 ± 1 °C and stored long term in Luria–Bertani (LB, Laboratorios Conda S.A., Madrid, Spain) broth with 20% glycerol at −80 °C. For the inoculum preparation, single colonies from 24 h old cultures were transferred in LB broth for 24 h at 27 ± 1 °C in a rotary shaker (180 rpm). Bacterial cultures were centrifuged at 5000× *g* for 15 min, and after discarding the supernatant, the pellets containing the bacterial cells were resuspended in sterile water. The density of the suspensions was normalized to an OD_600_ of 0.1, containing approximately 10^8^ colony-forming units (cfu)·mL^−1^ [71]. The SynCom MIX2 was assembled by mixing the bacterial suspensions of each strain in equal proportions (1:1:1…).

### 4.3. PEG-Induced Drought Stress in Arabidopsis thaliana

Seeds of *A. thaliana* ecotype Columbia were surface sterilized with 70% ethanol solution (10 min), 95% ethanol solution (10 min) [80], and then rinsed in sterile water five times. The seeds were then soaked for 30 min in the bacterial suspensions (sterile water for the negative control) and dried on sterile paper under laminar flow. Treated seeds were plated on square plates (120 mm × 120 mm) containing 0.5× Murashige and Skoog medium (MS) (Duchefa Biochemie, Haarlem, The Netherlands) supplemented with 0.3% gelrite (Duchefa Biochemie, Haarlem, The Netherlands) and 0.06% β-(N-Morpholino)ethanesulphonic acid (MES) monohydrate (Duchefa Biochemie, Haarlem, The Netherlands), with a pH of 5.8, and stratified for 48 h at 4 °C. The plates were transferred to a growth chamber under constant light conditions (23 °C, 80–100 µmol∙m^−2^∙s^−1^). Seven-day-old seedlings were transplanted to plates with lowered water potential (−1.2 MPa) or control plates with the same water potential (−0.25 MPa) prepared as previously described [59,81,82]. Briefly, water potential was lowered by infusing the agar plates with 550 g∙L^−1^ of PEG 6000 (polyethylene glycol, Sigma Aldrich, Stockholm, Sweden) overnight before use. After 24 h of growth, eight seedlings per plate were harvested for each treatment, with three plates per treatment.

#### Xylem Morphology Quantification

For the analysis of xylem morphology, roots were mounted directly in chloralhydrate solution, 8:2:1, chloralhydrate/glycerol/water (*w*/*v*/*v*), and visualized as described previously [59] using a Zeiss Axioscope A1 microscope (Jena, Germany) at 40× magnification with differential interference contrast (DIC) optics. For the quantification of phenotypes, the entire primary root was analyzed for differences from the typical pattern, separately for the distinct xylem axis positions: protoxylem (px), outer metaxylem (omx), and inner metaxylem (imx). Approximately 10 observations were conducted for each plant in order to cover the entire length of the primary root. The phenotypes were categorized, and the number of observations displaying a certain phenotype was used to calculate the frequency of the phenotype on the total observations.

### 4.4. Water Stress Induced by Water Deficit in Greenhouse

#### 4.4.1. Experiment Setup

Tomato seedlings (*Solanum lycopersicum* L. cv “Pixel”) were produced under standard conditions in a nursery in Ragusa, Italy. The seedlings were transplanted in a commercial greenhouse located in the same province (36°5103.2400 N 14°27041.4000 E) in soilless coconut fiber bags (KOLTURA, Itasmart S.R.L., Ragusa, Italy) (Supplementary Figure S3). The following treatments were tested in the trial: (a) control (C); (b) water stress (W); (c) *B. velezensis* PSE31B + water stress (W + 31B); (d) *P. salmasensis* POE54 + water stress (W + POE54); (e) MIX2 + water stress (W + MIX2). The strains *B. velezensis* PSE31B and *P. salmasensis* POE54 were selected based on growth promotion activity in tomato nursery plantlets [55]. Bacterial treatments were applied to the plants just before the transplant, submerging the roots for 30 min in the bacterial suspensions and 15 days later by soil drenching (50 mL per plant). Plants of the control and water stress theses were treated with water. Water stress was induced 24 h after the second treatment by reducing the irrigation application level by 40%. Briefly, during the first month, control plants were irrigated with 150 mL of water per plant, while stressed plants received 90 mL of water per plant. Later on, the irrigation volume was increased to 200 mL per plant for the controls and 120 mL for stressed plants. Nutrient management was consistent with local commercial practices. Basic fertilization with an NPK 9-18-27 fertilizer was used through the trial. Twelve plants for each treatment were used (three plants per coconut fiber bag). The experiment was performed using a completely randomized block design, with one coconut fiber bag representing one block (Supplementary Figure S3).

#### 4.4.2. Growth and Yield Attributes of Tomato Plants

Plant height, stem diameter, and shoot dry matter were recorded at the harvest (T8, eight weeks after stress application, 75 days after transplant). Plant height was also measured at T0 (after the transplant, before stress application) and T1 (a week after stress application) in order to calculate the growth rate, expressed as the height increase from T0 and T1 to T8 (end of the trial). The percentage of flowering plants on the total plants was monitored for up to six weeks after stress application (T6). The number of fruits per plant and the fruit weight were recorded at the harvest for the different trusses, as well as the number and weight of marketable fruits in the first truss. Twelve plants from each treatment were analyzed.

#### 4.4.3. Determination of Quality Parameters in Tomato Fruits

Tomato fruit firmness was measured at 23 °C using a non-destructive firmness device (Acoustic Firmness Sensor, Aweta Company, Pijnacker, The Netherlands). Measurements were performed by applying a compressive force to induce deformation of up to 2 mm along the equatorial axis of the fruit. A total of 12 fruits were analyzed per replicate.

The total soluble solids (TSS) were measured according to Seyoum et al. (2011) [83]. For each replicate, tomato juice from three tomatoes was extracted using a Kenwood citrus juicer (model JE290, Bolton, UK) and 50 mL of the resulting slurry was filtered through one layer of filter paper. The TSS content was determined using a refractometer (Atago Co., Ltd., model PR-32 α, Tokyo, Japan) by applying 1–2 drops of clarified juice to the prism. Between samples, the prism was rinsed with distilled water and dried. The refractometer was calibrated with distilled water (0 °Brix TSS). Three replicates per treatment were analyzed.

The titratable acidity (TA) of the tomatoes was evaluated according to the method described by Teerachaichayut and Ho (2017) [84], with the following slight modifications. For each replicate, juice from three tomatoes was extracted using a Kenwood juice extractor, and the resulting pulp was filtered. A 100 mL aliquot of the filtrate was centrifuged for 15 min, and the supernatant (clear juice) was used for analysis. Titratable acidity, expressed as the percentage of citric acid, was determined by titrating 10 mL of tomato juice to a pH of 8.2 with 0.1 M NaOH, using phenolphthalein as an indicator. The titratable acidity was calculated according to the following equation:

TA (% citric acid) = 0.0064 ∙ (mL of NaOH) ∙ 10

The sugar–acid ratio was calculated by dividing the total soluble solid by the titratable acidity of the given sample under analysis as described by Mohammed et al. (1999) [85] (nondimensional). Three replicates per treatment were analyzed.

### 4.5. Effect of Water Stress and Bacterial Treatments on Rhizosphere Bacterial Communities

#### 4.5.1. Sample Collection and Processing

Root rhizosphere samples were collected at the end of the trial (T8). Plants were cut at the collar and used for the phenotypic measurements. The coconut fiber bags were completely opened (Supplementary Figure S4), and portions of the root system and firmly attached substrate were taken, removing the bigger coconut fiber fragments, thus forming three bulk rhizosphere samples of four plants for each treatment (one random plant was chosen from each coconut fiber bag). Five grams of bulk samples were taken and suspended in 20 mL of saline buffer in sterile tubes and vortexed for 2 min. Pellets containing bacteria were collected by centrifugation (20 min at 16,750× *g*) and stored at −80 °C until DNA extraction [71].

#### 4.5.2. DNA Extraction, Library Preparation, and Amplicon Sequencing

Genomic DNA was extracted using the DNeasy PowerSoil Pro Kit (Qiagen, Hilden, Germany) following the manufacturer’s instructions. DNA concentration and quality were measured with a NanoDrop 1000 spectrophotometer (Thermo Scientific, Wilmington, DE, USA). Library preparation and amplicon sequencing were performed at IGA Technology Services (Udine, Italy). To profile bacterial communities, the V3–V4 hypervariable region of the bacterial 16S rRNA gene was amplified using primers 16S-341F and 16S-805R [86]. To minimize interference from host DNA, peptide nucleic acid (PNA) clamping was implemented during the initial amplification step, selectively blocking the amplification of host chloroplast and mitochondrial 16S rRNA gene sequences. The sequencing of the 16S libraries was conducted on an Illumina NovaSeq6000 system (Illumina, San Diego, CA, USA) using a 300 bp paired-end configuration. The paired-end reads were subsequently merged to generate high-quality, full-length sequences, ensuring reliable and accurate taxonomic identification.

#### 4.5.3. Bioinformatics

The merging of forward and reverse reads, quality filtering, and trimming, and the generation of Amplicon Sequence Variants (ASVs) were conducted using DADA2 (v. 1.26.0) [87] in R (v. 4.0.2) [88]. The taxonomic assignment of ASVs was carried out using the 16S SILVA 138 database [89] for 16S reads. ASVs related to plants as well as unassigned ASVs were removed from the 16S ASV table. α and β diversity analyses were conducted using the phyloseq package (v. 3.17) in R (v. 4.0.2). α diversity was estimated using the Chao1 richness and Shannon diversity indices to evaluate the diversity within each sample, and the Kruskal–Wallis test was used to assess the statistical significance. β diversity was evaluated using Bray–Curtis dissimilarity and visualized through Principal Coordinate Analysis (PCoA) to explore diversity within and between sample groups. Statistical significance between groups was determined using the PERMANOVA test through the vegan package [90] in R. A differential abundance analysis was performed with DESeq2 (v. 1.40.2) [91] to identify enriched or depleted bacterial ASVs in the rhizosphere of bacteria-treated plants compared to the plants subjected to water stress, considering the adjusted *p*-values (<0.05, False Discovery Rate, FDR).

#### 4.5.4. Statistical Analysis

ANOVA was used to analyze the data from the greenhouse trial using Minitab 20 statistical software (Minitab, Inc., State College, PA, USA). Means were separated using Fisher’s LSD test at a significance level of *p* ≤ 0.05.

## Figures and Tables

**Table 1:** Effects of water stress on the growth attributes of tomato plants in the greenhouse. Bacterial treatments were applied at the time of transplant and two weeks later by soil drenching. Stress was induced after the second bacterial treatment by reducing the irrigation regime by 40%. Twelve plants were measured for each treatment.

Treatment	Height			Stem Diameter (cm)		Dry Matter (%)
	T8 (cm)	T8-T0 (%)	T8-T1 (%)	T8 (2nd Truss)	T8 (4th Truss)	
WS	134.63 b	455.20 b	258.40 b	8.00 b	7.25 bc	12.01 a
WS_MIX2	148.78 ab	602.90 a	334.50 a	8.58 b	7.03 c	14.66 a
WS_PSE31B	160.56 a	555.70 a	329.94 a	8.81 b	8.21 b	14.19 a
WS_POE54	150.22 a	608.60 a	344.20 a	8.61 b	8.08 bc	13.51 a
Control	160.83 a	586.30 a	337.00 a	11.07 a	9.37 a	13.67 a
*p* value ^a^	<0.01	<0.05	<0.05	<0.001	<0.001	0.219

WS, water stress; WS_MIX2, water stress + MIX2; WS_PSE31B, water stress + *B. velezensis* PSE31B; WS_POE54, water stress + *P. salmasensis* POE54. ^a^ values (mean) in the same column followed by the same letter are not significantly different at *p* = 0.05 according to Fisher’s protected least significant difference.

**Table 2:** Effects of water stress on the yield attributes of tomato plants in the greenhouse. Bacterial treatments were applied at the time of transplant and two weeks later by soil drenching. Stress was induced after the second bacterial treatment by reducing the irrigation regime by 40%. Twelve plants were analyzed for each treatment.

Treatment	Flowering Plants (%)			Fruits							
	T3 (2nd Truss)	T5 (3rd Truss)	T6 (4th Truss)	T8 (1st Truss)		T8 (1st Truss, Marketable)		T8 (2nd Truss)		T8 (3rd Truss)	
				N.	Weight/ Fruit (g)	N.	Weight/ Fruit (g)	N.	Weight/ Fruit (g)	N.	Weight/ Fruit (g)
WS	91.67 a	75.00 a	66.67 a	30.25 a	21.31 a	20.50 a	23.45 a	22.50 a	18.30 a	25.25 a	11.93 a
WS_MIX2	91.67 a	91.67 a	91.67 a	29.00 a	20.37 a	19.00 a	23.10 a	24.75 a	18.64 a	26.67 a	11.14 a
WS_PSE31B	100.00 a	91.67 a	75.00 a	28.25 a	22.72 a	21.50 a	24.50 a	25.50 a	19.30 a	23.00 a	12.24 a
WS_POE54	91.67 a	83.33 a	91.67 a	30.33 a	19.35 a	18.00 a	22.90 a	24.33 a	18.03 a	24.00 a	11.05 a
Control	100.00 a	100.00 a	100.00 a	27.50 a	23.71 a	20.50 a	24.94 a	26.75 a	20.62 a	26.25 a	14.30 a
*p* value ^a^	0.736	0.252	0.221	0.818	0.091	0.322	0.477	0.473	0.486	0.417	0.103

WS, water stress; WS_MIX2, water stress + MIX2; WS_PSE31B, water stress + *B. velezensis* PSE31B; WS_POE54, water stress + *P. salmasensis* POE54. ^a^ values (mean) in the same column followed by the same letter are not significantly different at *p* = 0.05 according to Fisher’s protected least significant difference.

**Table 3:** Effects of water stress on the quality parameters of tomato fruits.

Treatment	Firmness (kg/cm^2^) ^a^	TSS (°Brix) ^b^	TA (g/L) ^b^	Sugar–Acid Ratio (TSS/TA) ^b^
WS	3.57 bc	5.77 a	0.53 c	10.99 a
WS_MIX2	3.25 bc	6.07 a	0.77 a	7.86 c
WS_PSE31B	3.65 b	6.07 a	0.70 ab	8.88 bc
WS_POE54	4.32 a	6.03 a	0.58 bc	10.52 ab
Control	3.13 c	6.00 a	0.56 bc	10.74 ab
*p* value ^c^	<0.0001	0.741	0.020	0.029

WS, water stress; WS_MIX2, water stress + MIX2; WS_PSE31B, water stress + *B. velezensis* PSE31B; WS_POE54, water stress + *P. salmasensis* POE54. ^a^ (*n* = 12). ^b^ (*n* = 3). ^c^ values (mean) in the same column followed by the same letter are not significantly different at *p* = 0.05 according to Fisher’s protected least significant difference.

## Data Availability

The raw data supporting the conclusions of this article will be made available by the authors on request.

## Supplementary Materials

The following supporting information can be downloaded at: https://www.mdpi.com/article/10.3390/ijms26041467/s1.
