# Supplementary material for: Mitigating Water Stress in Plants with Beneficial Bacteria: Effects on Growth and Rhizosphere Bacterial Communities
Source: Int J Mol Sci. 2025 Feb 10;26(4):1467. doi: 10.3390/ijms26041467 (PMC11855071; doi:10.3390/ijms26041467)
Supplement: Supplementary file 1 [file ijms-26-01467-s001.zip › Supplementary figures_Nicotra et al.pdf]

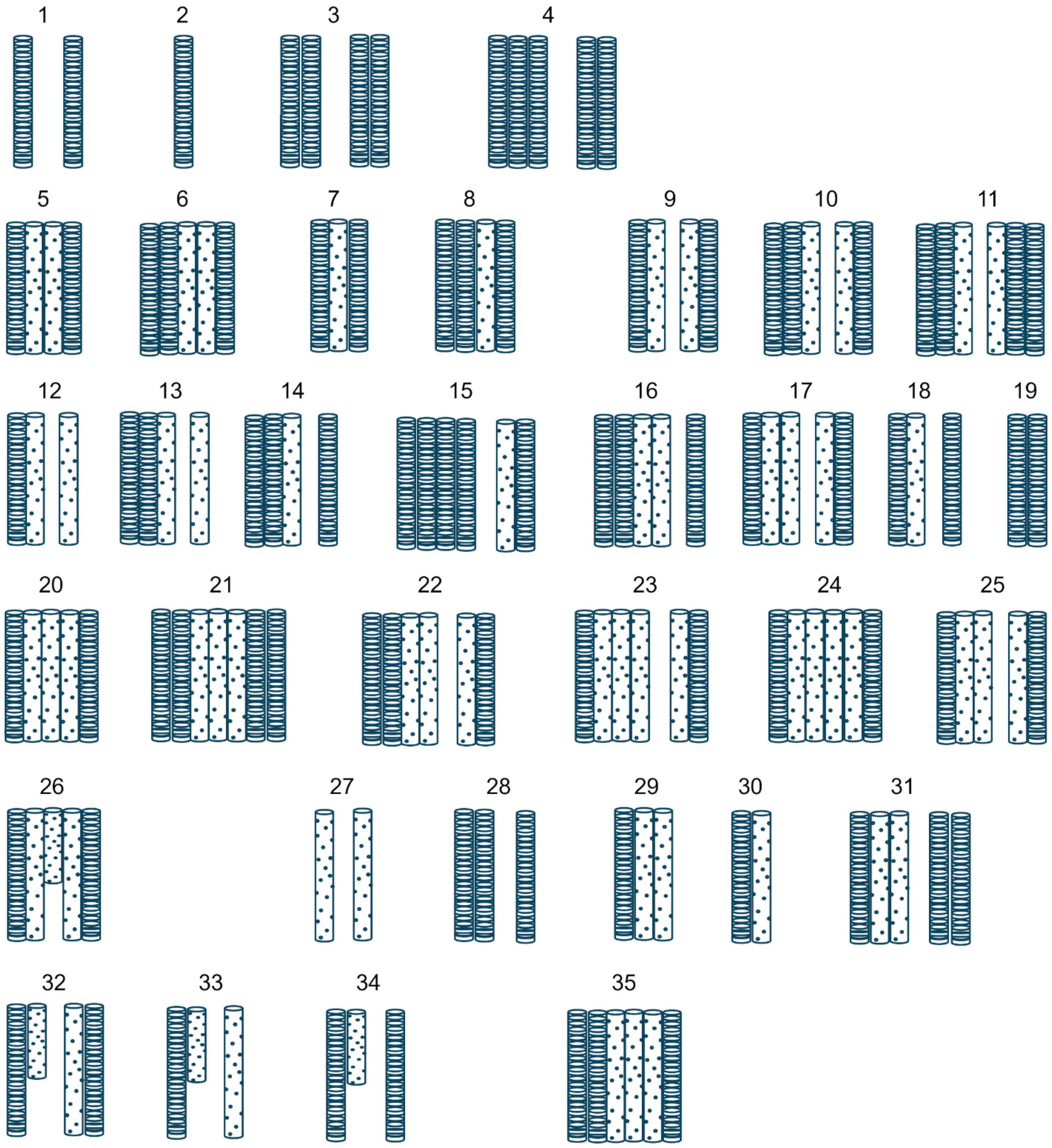

Figure S1: Root xylem phenotypes observed under microscope in *A. thaliana* seedlings exposed to lowered water potential (-1.2 MPa) in PEG-infused agar plates. Spiral strands represent protoxylem vessel. Pitted strands represent metaxylem vessels.

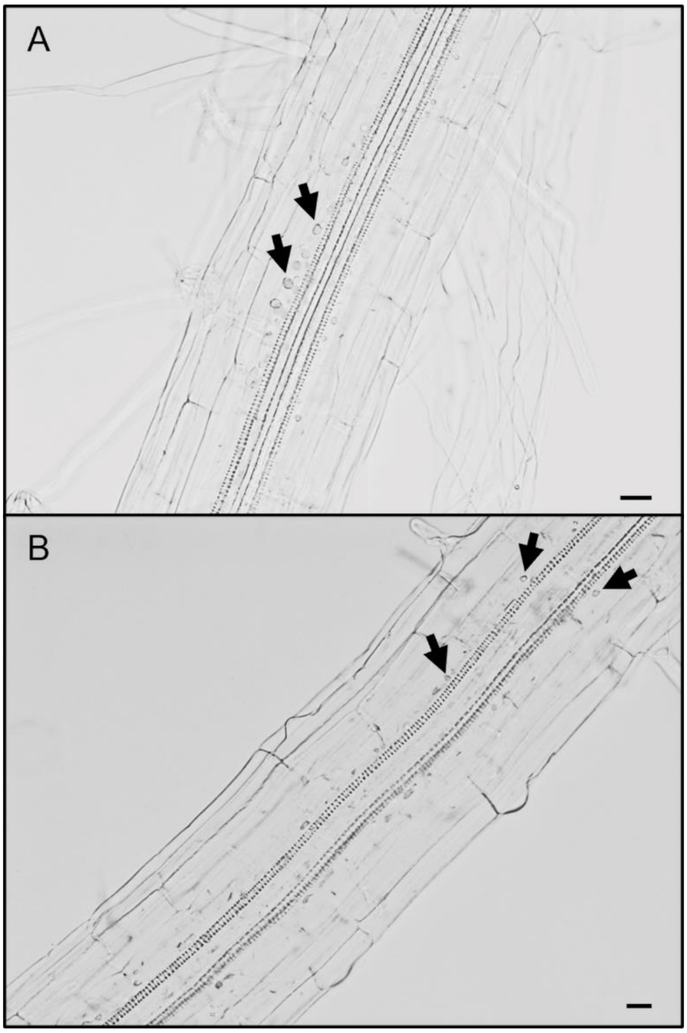

Figure S2: Other alterations of the normal phenotypes observed in *Arabidopsis* seedlings exposed to drought stress. (A) Presence of tyloses adjacent to protoxylem strands. (B) Presence of circular bodies adjacent to protoxylem strands. Scale bars = 10 µm.

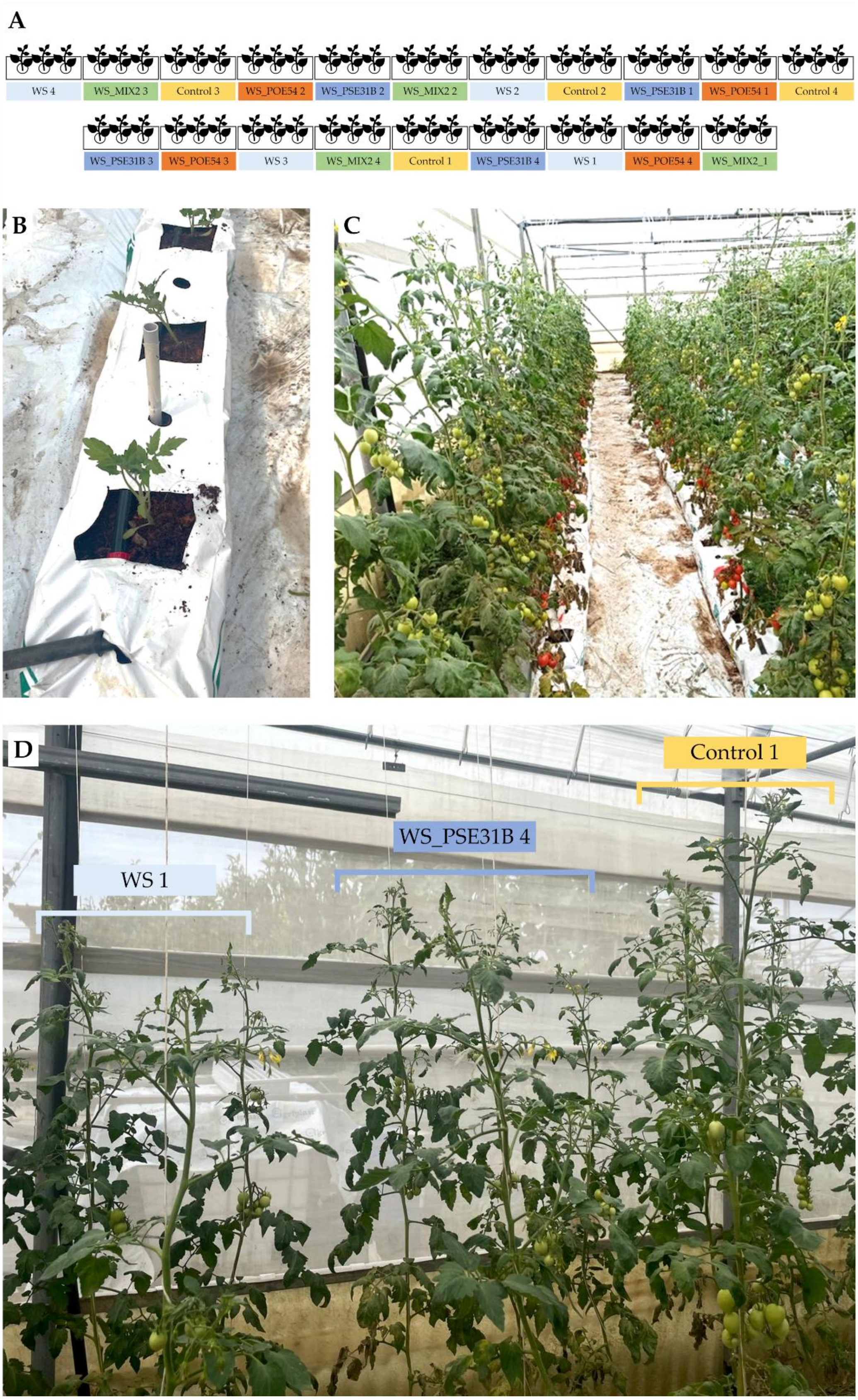

Figure S3: (A) Randomized block design of the greenhouse trial. (B) Coconut fiber bag with three tomato seedlings at the time of transplant (one block). (C) Tomato plants at the end of the trial (T8). (D) Differences in height between untreated water stressed plants, bacteria-treated water stressed plants and non-stressed control plants (from left to right).

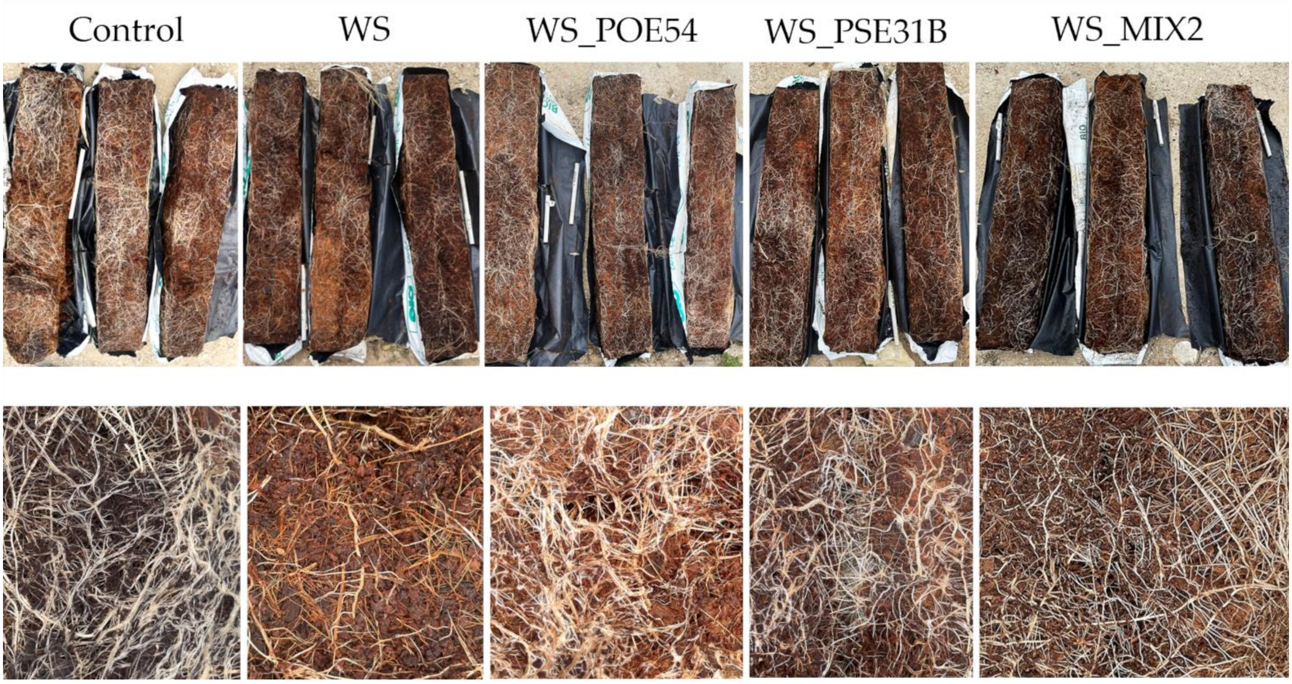

Figure S4: Coconut fiber bags opened at the end of the trial (top). Details of roots of the different treatments (bottom). Control, no stress; WS, water stress; WS_POE54, water stress + *P. salmasensis* POE54; WS_PSE31B, water stress + *B. velezensis* PSE31B; WS_MIX2, water stress + MIX2.

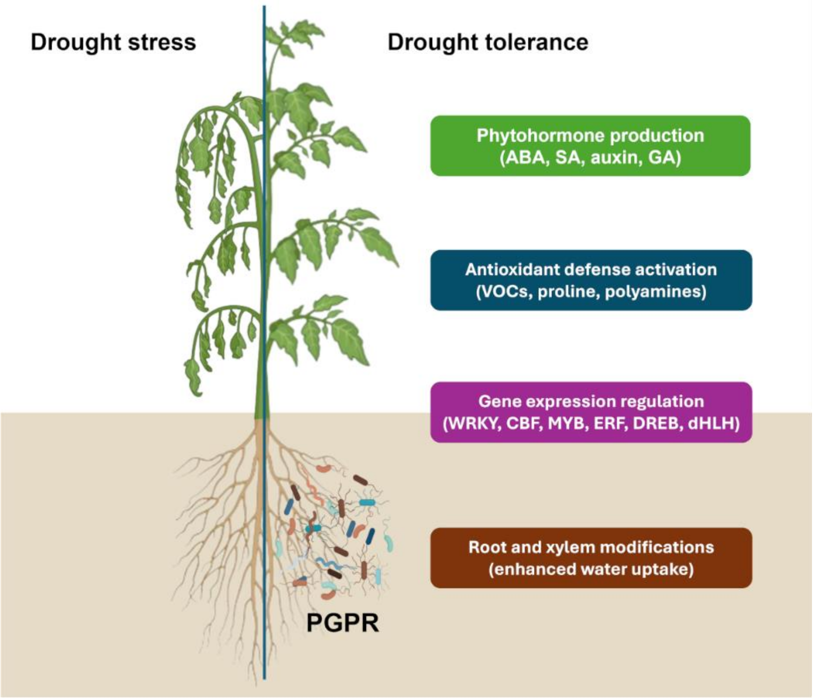

Figure S5: Coconut Regulatory Pathway of PGPR-Mediated Drought Stress Mitigation. Beneficial bacteria (PGPR) enhance drought tolerance through multiple mechanisms: (i) regulation of production of phytohormones like abscisic acid (ABA), salicylic acid (SA), auxin and gibberellic acid (GA) to regulate plant growth; (ii) activation of antioxidant defenses such as volatile organic compounds (VOCs), proline, and polyamines to reduce oxidative damage; (iii) root and xylem modifications to enhance water uptake efficiency; (iv) transcriptional regulation by stress-responsive transcription factors (WRKY, CBF, MYB, ERF, DREB, dHLH) to trigger adaptive stress responses.
